# Signs and symptoms in spatial neglect syndrome: a systematic review and meta-analysis

**DOI:** 10.1055/s-0046-1827209

**Published:** 2026-09-08

**Authors:** Tiago Costa da Silva, Davi Telécio Firmino, Camilla Vanessa Araújo Soares, Adriana M. T. Nepomuceno, Alex T. Meira

**Affiliations:** 1 Universidade Federal da Paraíba, Departamento de Medicina Interna, Serviço de Neurologia, João Pessoa PB, Brazil.; 2 Universidade Federal da Paraíba, Programa de Mestrado Profissional em Gerontologia, João Pessoa PB, Brazil.

**Keywords:** Perceptual Disorders, Stroke, Neurologic Manifestations, Spatial Behavior

## Abstract

**Background:**

Stroke is the second leading cause of mortality in the general population. Spatial neglect syndrome is one of the most common complications following stroke and is associated with poor prognosis, including prolonged hospitalization.

**Objective:**

To examine the main clinical signs and symptoms associated with spatial neglect in stroke patients in the literature.

**Methods:**

The present systematic review and meta-analysis was conducted following the Preferred Reporting Items for Systematic Reviews and Meta-Analyses protocol. The Grading of Recommendations Assessment, Development and Evaluation approach was applied to assess the certainty of evidence, and the data were processed with RevMan Web (The Cochrane Collaboration).

**Results:**

A total of 362 articles were assessed, out of which 23 were included. A strong correlation was observed between clinical events and the presence of spatial neglect syndrome compared to control groups (oculomotor exploratory behavior [ *p* < 0.00001; 95% CI 4.03–22.41], difficulty detecting stimuli on the left side ( *p* < 0.0001; 95% CI 3.20–24.89), and prolonged reaction time to left-sided stimuli ( *p* = 0.004; 95% CI 1.11–273.20). The present study confirms that spatial neglect syndrome presents as a clinically diverse condition. The quantitative analysis demonstrated that abnormal oculomotor behavior is the most robust and consistent clinical predictor for diagnosis. Other frequent symptoms included delayed reaction times and impaired detection of stimuli in the left visual field. Additionally, the presence of hemianopia and the crossed legs sign were also associated with spatial neglect syndrome among others.

**Conclusion:**

The symptomatology described may serve as an indication of spatial neglect syndrome. Therefore, the present meta-analysis highlights the clinical importance of early identification and targeted evaluation in stroke cases.

## INTRODUCTION

Stroke is the second leading cause of mortality in the general population, and its incidence is expected to rise due to population aging. 1 A frequent complication of right middle cerebral artery stroke is spatial neglect syndrome. 2 This syndrome is a complex attentional disorder toward the space contralateral to the lesion. It most commonly presents as unilateral neglect (80%), with a higher prevalence following right hemisphere lesions, leading to left-sided spatial perception deficits. Conversely, neglect affecting the right space is rare. Patients with neglect may exhibit a range of deficits, including personal (e.g., attending to only one side of the body during hygiene, failing to recognize contralateral body parts as their own), auditory (e.g., not responding to sounds from one side despite intact hearing), visual (e.g., ignoring stimuli in the left visual field), and tactile impairments (e.g., failing to respond to tactile input on one side of the body). 3 Notably, this process is unconscious to the patient. 4 Spatial neglect is associated with poor prognosis, including prolonged hospitalization and increased complications, warranting early diagnosis. 2 Additionally, it is associated with conditions known to impair the vertical field of view, such as pusher syndrome, as well as a broader range of signs, including the crossed legs sign. 4 5 6

The onset is typically in the acute phase of stroke but may persist as a chronic deficit. Major complications associated with spatial neglect syndrome include an elevated risk of falls, reduced autonomy, worsened motor impairments, mood disorders, and delusions. 2 7 8 In clinical practice, patients rarely report spatial neglect syndrome symptoms spontaneously, underscoring the importance of thorough history-taking and physical examination. 2

Spatial neglect syndrome is one of the most common stroke complications and numerous studies have assessed diagnostic tools for its evaluation. The present study aims to examine the main clinical signs and symptoms associated with spatial neglect in stroke patients. Specifically, it seeks to discuss the key symptoms features of spatial neglect syndrome as reported in the literature following stroke.

## METHODS

The present systematic review and meta-analysis was conducted following the Preferred Reporting Items for Systematic Reviews and Meta-Analyses (PRISMA) guidelines. 9

### Information sources and search strategy

The literature search was performed in the following electronic databases: U.S. National Library of Medicine (PubMed), Latin American and Caribbean Literature in Health Sciences (LILACS), and Embase. Controlled vocabulary terms were identified using the Health Sciences Descriptors (DeCS) and the Medical Subject Headings (MeSH), with the descriptors *Spatial Neglect* AND *Signs* *and* *Symptoms* .

The research question was structured using the PICOTT framework, as follows:

P (population): Patients with spatial neglect syndrome.
I (intervention): Evaluation during diagnosis or treatment involving one or more signs and symptoms of spatial neglect syndrome.
C (comparison): Absence of signs of spatial neglect syndrome.
O (outcome): Identified visual, tactile or auditory signs and symptoms of spatial neglect syndrome
T (type of study): Observational and randomized or non-randomized clinical trial.
T (type of question): Undetermined.

**Figure 1:**
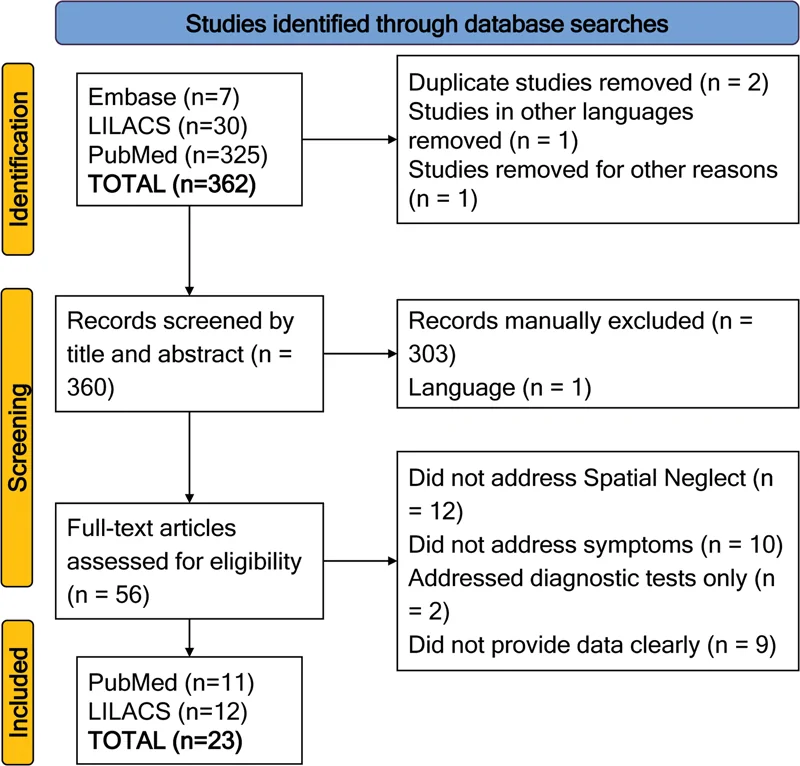
Source: Survey data. Prisma flow diagram of the study. **Notes** : A total of 362 records were identified through database searches (PubMed, LILACS, and Embase). After removing duplicates and ineligible studies, 56 full-text articles were assessed for eligibility. Twenty-three studies met the inclusion criteria and were included in the systematic review.

Based on this structure, the guiding research question was: “What are the most relevant signs and symptoms in the clinical presentation of spatial neglect syndrome?” The complete search strategies for each database are presented in Figure 1 . The authors conducted the searches between January and February 2025.

### Eligibility criteria and study selection

Eligible studies were primary investigations published in the last 10 years, focusing on signs and symptoms associated with spatial neglect. The inclusion criteria encompassed observational and interventional studies. Exclusion criteria comprised review articles, case reports, studies unrelated to the topic, and those published in languages other than English or Portuguese ( Figure 1 ).

After removing duplicates, two independent reviewers screened the titles and abstracts. Articles deemed potentially relevant were retrieved in full and assessed for eligibility. Any disagreements were resolved through discussion or by consulting a third reviewer. The final selection of studies was submitted to descriptive synthesis, and data were organized in structured tables for further analysis and interpretation ( Figure 1 ).

### Data collection process and data items

Data extraction was independently performed by two reviewers using a standardized Microsoft Excel (Microsoft Corp.) spreadsheet. The following variables were extracted: bibliographic information (first author, year of publication, country); methodological characteristics (study design, sample size, mean age, sex distribution); and main outcomes.

### Risk of bias analysis

Risk of bias was assessed using two tools: for non-randomized studies, the Risk of Bias in Non-randomized Studies – of Interventions (ROBINS-I) tool was used ), and for randomized studies, the Risk of Bias in Randomized Trials – Version 2 (RoB2) tool was used.

### Statistical analysis and certainty of evidence

A quantitative descriptive analysis was performed, summarizing the sample size, mean age, and sex distribution of participants across the studies included. A qualitative synthesis was also performed to categorize the types of signs and symptoms reported, as well as the subtypes of spatial neglect identified.

For studies with sufficient data, a random-effects meta-analysis was performed focusing on the three most frequently reported outcomes, considered as key indicators of spatial neglect syndrome. These outcomes were analyzed to estimate pooled event rates and heterogeneity.

To assess the certainty of evidence, the Grading of Recommendations Assessment, Development and Evaluation (GRADE) approach was applied using the GRADEpro GDT (Evidence Prime Inc. and McMaster University) platform. Statistical analyses were performed using RevMan Web (The Cochrane Collaboration).

## RESULTS

Twenty-three studies were analyzed for the preparation of the current systematic review ( Figure 1 ). The epidemiological profile of individuals with spatial neglect syndrome revealed that most participants were male with a mean age of 60 years. The most prevalent type of spatial neglect was visual. The study with the largest sample size (n = 101) was conducted by Nelemans et al., 10 while the study with the smallest sample (n = 19) was conducted by Làdavas, Tosatto, and Bertini. 11 Descriptive statistical analysis data are presented in **Supplementary Material Table S1** (available at https://www.arquivosdeneuropsiquiatria.org/wp-content/uploads/2026/05/ANP-2025.0389-Supplementary-Material.docx ).

**Figure 2:**
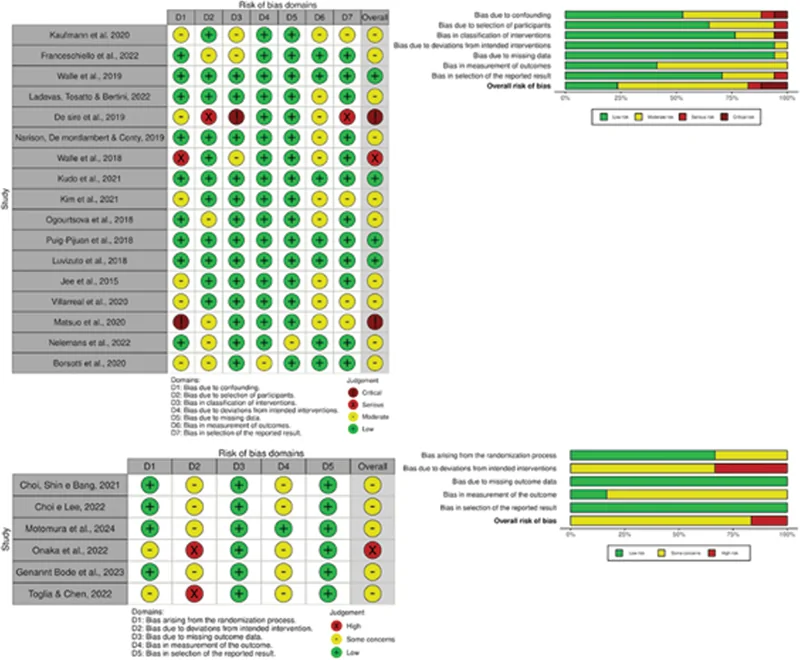
Source: Survey data. Risk of bias analysis conducted with the Risk of Bias in Non-randomized Studies – of Interventions (ROBINS-I) and Risk of Bias in Randomized Trials – Version 2 (RoB 2) tools. **Notes** : The upper panel shows non-randomized studies evaluated using the ROBINS-I tool, assessing bias across seven domains: confounding factors, participant selection, classification of interventions, deviations from intended interventions, missing data, measurement of outcomes, and selection of the reported result. The lower panel presents randomized controlled trials assessed with the RoB 2 tool, evaluating five domains: randomization process, deviations from intended interventions, missing outcome data, measurement of outcomes, and selection of reported results. Green indicates low risk, yellow indicates some concerns, and red indicates high risk of bias.

Among the studies evaluated using ROBINS-I, four were classified as low risk of bias, 12 13 14 demonstrating methodological rigor. Ten studies were considered moderate-risk, presenting limitations yet providing some level of reliable evidence. One study 15 was rated as serious risk of bias, and two 16 17 as critical risk, indicating significant methodological flaws. The ROBINS-I assessment is illustrated in Figure 2 . For the studies assessed with RoB 2, none was classified as low risk. Five studies 18 19 20 21 22 were rated as moderate risk of bias, due to concerns such as selection bias and outcome measurement. One study 23 presented a high risk of bias. The RoB 2 assessments are shown in Figure 2 .

Visual spatial neglect is the most frequent outcome which included symptoms as hypokinesia of gaze directed to the left, reduced detection of stimuli, particularly on the left side, among others. 12 15 19 24 25 26 Difficulty in detecting stimuli on the left side emerged as the second most prevalent outcome, identified in the studies by Narison, De Montalembert, and Conty, 26 Ogourtsova et al., 27 Walle et al. 24 and Toglia and Chen. 17 A prolonged reaction time to left-sided stimuli was the third most prevalent outcome, identified in the studies by Ogourtsova et al., 27 Villarreal et al. 28 and Motomura et al. 21

Other outcomes identified in the studies included reduced pupillary dilation, more impaired detection of ipsilateral stimuli, poorer oral hygiene on the left side, directional hypokinesia of gaze with increased fixation time on the right side, anosognosia for the left side deficit, greater attentional lateralization deficit when exposed to a high variety of stimuli, hemianopia as an aggravating factor of neglect, reduced mobility in patients with neglect, impaired leftward head movement, rightward head deviation, impaired auditory localization on the left side and for distant sounds, worsened neglect in the seated position, and the crossed-leg sign. 10 11 14 15 16 17 19 20 23 30 31 The outcomes are detailed in **Supplementary Material Table S1** .

**Figure 3:**
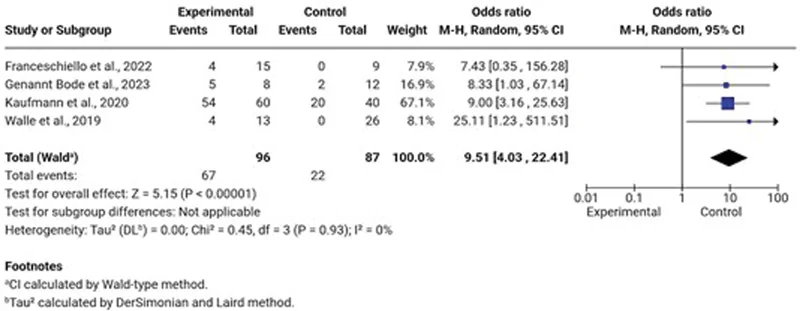
Source: Survey data. Analysis of the outcome *rightward oculomotor exploration behavior* . **Notes** : Forest plot of the meta-analysis comparing the experimental and control groups regarding the studied outcome. No heterogeneity was observed (I ^2^ = 0%). The overall analysis demonstrated a significantly higher odds of events in the experimental group compared to the control group (odds ratio = 9.51; 95% confidence interval, 4.03–22.41; *p* < 0.00001).

**Figure 4:**
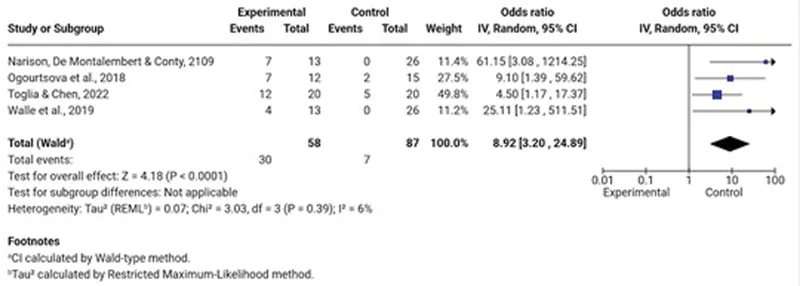
Source: Survey data. Analysis of the outcome *increased left-sided stimulus reaction time* . **Notes** : Forest plot of the meta-analysis comparing the experimental and control groups regarding the studied outcome. Low heterogeneity was observed (I ^2^ ¼ 6%). The overall effect indicated significantly higher odds of events in the experimental group compared to controls (odds ratio ¼ 8.92; 95% confidence interval, 3.20–24.89; *p* < 0.0001).

**Figure 5:**
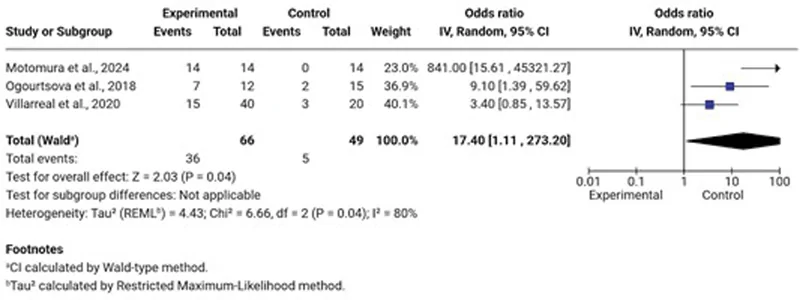
Notes: Forest plot of the meta-analysis comparing the experimental and control groups for the variable studied. High heterogeneity was observed (I ^2^ = 80%). The overall analysis demonstrated a significant difference favoring the experimental group (OR = 17.40; 95% CI, 1.11–273.20; *p* = 0.04). Analysis of the outcome *prolonged reaction time to left-sided stimuli* .

Regarding outcome data analysis, three outcomes were analyzed: rightward oculomotor exploration behavior, increased left-sided stimulus reaction time and impaired detection of left-sided stimuli. A strong correlation was observed for oculomotor behavior as a spatial neglect syndrome symptom ( *p* < 0.00001; CI [4.03–22.41]; odds ratio = 9.51; Tau ^2^ = 0.0; Chi ^2^ = 0.45; I ^2^ = 0%) ( Figure 3 ) and for impaired detection of left-sided stimuli ( *p* < 0.0001; CI [3.20–24.89]; odds ratio = 8.92; Tau ^2^ = 0.07; Chi ^2^ = 3.03; I ^2^ = 6%) ( Figure 4 ). For the outcome prolonged reaction time to left-sided stimuli, high heterogeneity was found, which may overestimate the odds ratio ( *p* = 0.004; CI [1.11–273.20]; odds ratio = 17.40; Tau ^2^ = 4.43; Chi ^2^ = 6.66; I ^2^ = 80%) ( Figure 5 ).

### Certainty of evidence analysis

The analysis considered the following outcomes: rightward oculomotor exploration behavior, increased left-sided stimulus reaction time and impaired detection of left-sided stimuli. The outcome of oculomotor behavior showed moderate certainty of evidence with an odds ratio of 9.51 (4.03–22.41) and an absolute effect indicating 79 out of 100 patients with this symptom in spatial neglect. The outcome of prolonged reaction time was classified as low certainty of evidence with an odds ratio of 17.40 (1.11–273) and an absolute effect of 66 out of 100 patients with neglect. Finally, the outcome of impaired detection stimuli also showed low certainty of evidence with an odds ratio of 8.93 (3.20–24.89) and an absolute effect of 44 out of 100 patients with neglect ( **Supplementary Material Table S2** ).

## DISCUSSION

The symptomatology of spatial neglect syndrome presents a diverse clinical manifestation, making its recognition relevant to medical practice in the care of post-stroke patients. In the 23 studies analyzed, this premise is confirmed by identifying signs and symptoms that range from ocular motricity to postural positioning in bed.

The outcome *oculomotor behavior* in the studies led by Kaufmann et al. 23 and Franceschiello et al. 25 shows that such behavior is more pronounced on the right side, manifesting as a more prominent exploration of the environment to the right. The work by Kudo et al. 12 demonstrates that oculomotor behavior is expressed through saccadic movements contralateral to the lesion (e.g., on the left side), characterized by slower and smaller movements and reduced detection of ipsilateral stimuli. Other forms of oculomotor behavior in patients with neglect include changes in pupillary response to visual stimuli showing reduced dilation (and no reaction to cognitive stimuli). Directional gaze hypokinesia, with longer fixation on the right side, is also observed in patients with neglect and is more intense in severe cases. Additionally, ipsilateral visual deficits in oculomotor behavior are described. 15 19 24 This was the outcome with the highest level of evidence classified as moderate, although not with absolute certainty of effect.

The outcome *difficulty detecting stimuli on the left side* can be understood as a reduced ability to identify and interpret a stimulus related to the spatial location of an object presented in the left visual field due to an attentional bias. It also includes the inability to consciously recognize this deficit in neglect. There is greater fixation on the right visual field and motor weakness towards the left visual field. 18 24 27 28 This outcome showed low certainty of evidence, though with good statistical consistency. However, methodological weaknesses in the studies may justify this level of evidence.

For the outcome *prolonged reaction time to left-sided stimuli* , when visual stimuli were presented in the left visual field, reduced detection was observed, especially in more complex environments or with increased cognitive load. This finding should be considered in treatment planning, indicating the need for gradual and adaptive stimulus progression tailored to the patient's condition. 22 28 29 30 This outcome was classified as having low certainty of evidence due to an elevated odds ratio with a wide confidence interval and a highly heterogeneous sample, suggesting that the observed effect may be rare; contradicting the findings of the studies analyzed and potentially limiting its applicability.

Other outcomes in the studies addressed different symptoms, such as the relationship between hemianopia and spatial neglect, in which the presence of this visual deficit may worsen symptoms in the long term 13 ; evidence of poorer oral hygiene on the left side 16 ; and the crossed-leg sign, a posture adopted in bed during the acute phase of stroke. 14 Other symptoms include reduced left-hand grip strength while writing, 31 worse mobility in some patients with neglect, characterized by impaired spatial orientation and increased time to complete walking tasks, 10 longer time to detect a stimulus located far in the left visual field, 32 impaired leftward head movement, 1 rightward head deviation as an aggravating factor of spatial neglect, 21 impaired auditory localization on the left side with perception of sounds as being farther than they are 17 and worsening of neglect symptoms when seated 23

In addition to these signs and symptoms identified in our study, other symptoms described in the literature include lower visual field deficits in the craniocaudal direction, acalculia for two-digit numbers and left-sided hemianopia. 33 34 35 However, since these are case reports, they lack scientific evidence and were not included in this work.

Given the outcome analysis, it is evident that the most frequently discussed type of neglect in the literature is visual spatial neglect. Also, there appear to be numerous diagnostic possibilities for this syndrome that can guide patient treatment. Another aspect observed was the applicability of video-oculography in spontaneous spatial exploration, which may be more sensitive for detecting neglect than traditional paper-and-pencil tests 25 as the traditional Behavior Inattentional Tasks (BIT)—although it may not be available in many hospitals which can limit its use as a diagnostic tool of spatial neglect syndrome. The study methodologies included tools such as virtual reality, cognitive tests, and audiovisual tests for diagnosing spatial neglect, highlighting a range of possibilities for investigating this syndrome in stroke patients presenting a wide range of possibilities to manage this syndrome.

In conclusion, the symptomatology described is diverse and may serve as an indication of spatial neglect syndrome in patients affected by stroke. Therefore, the current meta-analysis highlights the clinical importance of early identification and targeted evaluation in stroke cases. The most consistent and frequently reported symptoms were oculomotor behavior and impaired detection of stimuli in the left visual field. Moreover, this discussion has clinical applicability in supporting stroke assessment, considering that the persistence of spatial neglect syndrome may serve as a poor prognostic factor for recovery. Thus, this knowledge may contribute to rehabilitation efforts by enabling the early investigation of the issues discussed.

## References

[1] Xu W Zhang X Chen H Zhao Z Zhu M Prevalence and outcome of young stroke patients with middle cerebral artery stenosis BMC Neurol 2021 21 01 99 10.1186/s12883-021-02125-8 33663425 PMC7931598

[2] Sarwar A Emmady P D Spatial neglect [Internet] 2020[cited Nov 5, 2025]. Available from:https://www.ncbi.nlm.nih.gov/books/NBK562184/

[3] Lopes M L San'Anna MdS Junior Ferreira H P As diferentes manifestações da heminegligência e sua avaliação clínica Fisioter Bras 2018 19 02 10.33233/fb.v19i2.2314

[4] Bazan R Fernandes T Braga G Luvizutto G Resende L A new clinical sign probably associated to left hemiplegia with left hemineglect syndrome: the crossed legs" Arq Neuropsychiatr 2014 72 06 418 421 10.1590/0004-282x20140043 24964106

[5] Rémi J Pfefferkorn T Owens R L The crossed leg sign indicates a favorable outcome after severe stroke Neurology 2011 77 15 1453 1456 10.1212/WNL.0b013e318232abe4 21987641 PMC3198984

[6] Embrechts E van der Waal C Anseeuw D Association between spatial neglect and impaired verticality perception after stroke: A systematic review Ann Phys Rehabil Med 2023 66 03 101700 10.1016/j.rehab.2022.101700 35963568

[7] Giannakou I Lin D Punt D Computer-based assessment of unilateral spatial neglect: A systematic review Front Neurosci 2022 16 912626 10.3389/fnins.2022.912626 36061603 PMC9437703

[8] Moore M Milosevich E Beisteiner R Rapid screening for neglect following stroke: A systematic search and European Academy of Neurology recommendations Eur J Neurol 2022 29 09 2596 2606 10.1111/ene.15381 35510782 PMC9544365

[9] PRISMA Group Moher D Liberati A Tetzlaff J Altman D G Preferred reporting items for systematic reviews and meta-analyses: the PRISMA statement PLoS Med 2009 6 07 e1000097 10.1371/journal.pmed.1000097 21603045 PMC3090117

[10] KnowledgeBrokers Neglect Study Group Nelemans K N Nijboer T CW Brink A FT van der Stigchel S The mobility assessment course: A ready-to-use dynamic measure of visuospatial neglect J Neuropsychol 2022 16 03 498 517 10.1111/jnp.12277 35445544

[11] Làdavas E Tosatto L Bertini C Behavioural and functional changes in neglect after multisensory stimulation Neuropsychol Rehabil 2022 32 05 662 689 10.1080/09602011.2020.1786411 32602783

[12] Kudo Y Takahashi K Sugawara E Bedside video-oculographic evaluation of eye movements in acute supratentorial stroke patients: A potential biomarker for hemispatial neglect J Neurol Sci 2021 425 117442 10.1016/j.jns.2021.117442 33857735

[13] Puig-Pijoan A Giralt-Steinhauer E de Torres Az Underdiagnosis of unilateral spatial neglect in stroke unit Acta Neurol Scand 2018 138 05 441 446 10.1111/ane.12998 30058181

[14] Walle K M Nordvik J E Espeseth T Becker F Laeng B Multiple object tracking and pupillometry reveal deficits in both selective and intensive attention in unilateral spatial neglect J Clin Exp Neuropsychol 2019 41 03 270 289 10.1080/13803395.2018.1536735 30426866

[15] Sire Ad Baricich A Ferrillo M Migliario M Cisari C Invernizzi M Buccal hemineglect: is it useful to evaluate the differences between the two halves of the oral cavity for the multidisciplinary rehabilitative management of right brain stroke survivors? A cross-sectional study Top Stroke Rehabil 2020 27 03 208 214 10.1080/10749357.2019.1673592 31714187

[16] Matsuo T Moriuchi T Iso N Effects of prism adaptation on auditory spatial attention in patients with left unilateral spatial neglect: a non-randomized pilot trial Int J Rehabil Res 2020 43 03 228 234 10.1097/MRR.0000000000000413 32776764

[17] Toglia J Chen P Spatial exploration strategy training for spatial neglect: A pilot study Neuropsychol Rehabil 2022 32 05 792 813 10.1080/09602011.2020.1790394 32684100 PMC7855016

[18] Bode L KG Sprenger A Helmchen C Hauptmann B Münte T F Machner B Combined optokinetic stimulation and cueing-assisted reading therapy to treat hemispatial neglect: A randomized controlled crossover trial Ann Phys Rehabil Med 2023 66 05 101713 10.1016/j.rehab.2022.101713 36645965

[19] Choi H-S Shin W-S Bang D-H Application of digital practice to improve head movement, visual perception and activities of daily living for subacute stroke patients with unilateral spatial neglect: Preliminary results of a single-blinded, randomized controlled trial Medicine (Baltimore) 2021 100 06 e24637 10.1097/MD.0000000000024637 33578583 PMC7886475

[20] Choi H-S Lee B-M A Complex Intervention Integrating Prism Adaptation and Neck Vibration for Unilateral Neglect in Patients of Chronic Stroke: A Randomised Controlled Trial Int J Environ Res Public Health 2022 19 20 13479Retracted and replaced in: Int J Environ Res Public Health 2026;23(4):536 10.3390/ijerph23040536 10.3390/ijerph23040536 PMC13101745 42018285

[21] Motomura K Amimoto K Numao T Kaneko F Effects of a stimulus response task using virtual reality on unilateral spatial neglect: a randomized controlled trial Arch Phys Med Rehabil 2024 105 08 1449 1457 10.1016/j.apmr.2024.05.009 38750715

[22] Onaka H Kouda K Nishimura Y Standing and supine positions are better than sitting in improving rightward deviation in right-hemispheric stroke patients with unilateral spatial neglect: A randomized trial Medicine (Baltimore) 2022 101 46 e31571 10.1097/MD.0000000000031571 36401369 PMC9678496

[23] Kaufmann B C Cazzoli D Pflugshaupt T Eyetracking during free visual exploration detects neglect more reliably than paper-pencil tests Cortex 2020 129 223 235 10.1016/j.cortex.2020.04.021 32512414

[24] Walle K M Nordvik J E Becker F Espeseth T Sneve M H Laeng B Unilateral neglect post stroke: Eye movement frequencies indicate directional hypokinesia while fixation distributions suggest compensational mechanism Brain Behav 2019 9 01 e01170 10.1002/brb3.1170 30548825 PMC6346647

[25] Franceschiello B Noto T D Bourgeois A Machine learning algorithms on eye tracking trajectories to classify patients with spatial neglect Comput Methods Programs Biomed 2022 221 106929 10.1016/j.cmpb.2022.106929 35675721

[26] Narison R De Montalembert M Conty L Diagnosing gaze and arrow cueing effects in unilateral spatial neglect Neurocase 2020 26 01 42 50 10.1080/13554794.2019.1705495 31856672

[27] Ogourtsova T Archambault P Sangani S Lamontagne A Ecological virtual reality evaluation of neglect symptoms (EVENS): effects of virtual scene complexity in the assessment of poststroke unilateral spatial neglect Neurorehabil Neural Repair 2018 32 01 46 61 10.1177/1545968317751677 29357742

[28] Villarreal S Linnavuo M Sepponen R Vuori O Jokinen H Hietanen M Dual-Task in Large Perceptual Space Reveals Subclinical Hemispatial Neglect J Int Neuropsychol Soc 2020 26 10 993 1005 10.1017/S1355617720000508 32456748

[29] IRCCS Don Gnocchi Stroke Group Borsotti M Mosca I E Di Lauro F The Visual Scanning Test: a newly developed neuropsychological tool to assess and target rehabilitation of extrapersonal visual unilateral spatial neglect Neurol Sci 2020 41 05 1145 1152 10.1007/s10072-019-04218-2 31897939

[30] Jee H Kim J Kim C Kim T Park J Feasibility of a Semi-computerized Line Bisection Test for Unilateral Visual Neglect Assessment Appl Clin Inform 2015 6 02 400 417 10.4338/ACI-2015-01-RA-0002 26171084 PMC4493339

[31] Kim T-L Kim K Choi C Lee J-Y Shin J-H FOPR test: a virtual reality-based technique to assess field of perception and field of regard in hemispatial neglect J Neuroeng Rehabil 2021 18 01 39 10.1186/s12984-021-00835-1 33602254 PMC7890954

[32] Numao T Amimoto K Shimada T Examination and treatment of unilateral spatial neglect using virtual reality in three-dimensional space Neurocase 2021 27 06 447 451 10.1080/13554794.2021.1999478 34927563

[33] Jang Y Lee E Kim Y Park J H Han S Number Processing Error as a Clinical Manifestation of Hemispatial Neglect Following Hypoxic Brain Injury: a Case Report Brain Neurorehabil 2020 13 03 e20 10.12786/bn.2020.13.e20 PMC9879366 36741793

[34] Hamada Y Shigehisa A Kanda Y Enhancement of the Ivy Sign during an Ischemic Event in Moyamoya Disease Intern Med 2023 62 04 617 621 10.2169/internalmedicine.9326-22 35908969 PMC10017230

[35] Grattan E S Skidmore E R Woodbury M L Examining anosognosia of neglect OTJR (Thorofare, NJ) 2018 38 02 113 120 10.1177/1539449217747586 PMC5930481 29251546

